# Hepatoprotective Effects of Ethyl Acetate Extract of *Lygodium microphyllum* Leaves

**DOI:** 10.5812/ijpr-171722

**Published:** 2026-06-14

**Authors:** Hadi Kuncoro, Erna Muliana, Putri Anggreini, Angga Cipta Narsa, Zulhaerana Bahar, Vina Maulidya, Nurul Annisa

**Affiliations:** 1 Faculty of Pharmacy, Mulawarman University, Samarinda, Indonesia

**Keywords:** L. Microphyllum, Paracetamol, Aspartate Aminotransferase (AST), Alanine Aminotransferase (ALT)

## Abstract

**Background:**

*Lygodium microphyllum* is traditionally used in Chinese medicine as a decoction to treat hepatitis.

**Objectives:**

This study evaluated the in vivo hepatoprotective efficacy and optimal dose of the ethyl acetate extract of *L. microphyllum* leaves (EALM) against paracetamol-induced liver injury.

**Methods:**

A pilot experiment was conducted in 24 male Wistar rats divided into six groups: a normal control group, a paracetamol control group (3 g/kg body weight [BW]), and three EALM groups (200, 400, and 600 mg/kg BW). EALM was administered orally for 14 days, and paracetamol was administered once on day 8. The evaluated parameters included body weight, serum aspartate aminotransferase (AST) and alanine aminotransferase (ALT) levels, and liver histopathology. Paracetamol intoxication caused significant weight loss and increased AST and ALT levels (P < 0.0001).

**Results:**

All EALM doses significantly attenuated paracetamol-induced enzyme elevations. Histopathological analysis revealed severe injury, including pyknosis, in the paracetamol group, whereas EALM-treated livers maintained near-normal morphology. Among the tested doses, 200 mg/kg BW showed the most pronounced protective effect.

**Conclusions:**

These findings indicate that EALM has significant hepatoprotective activity against paracetamol-induced hepatotoxicity.

## 1. Background

Herbal treatments derived from natural sources are generally associated with a lower incidence of adverse effects than synthetic pharmaceuticals, contributing to an increasing public preference for plant-based medicine. Another advantage of natural compounds is their rich content of secondary metabolites. Research on liver injury has identified several of these metabolites, including triterpenoids, flavonoids, and polyphenols, as highly effective hepatoprotective agents (1).

*Lygodium microphyllum* is one such natural product with documented hepatoprotective potential. It has a history of traditional use in Chinese medicine, in which a decoction prepared from the plant is consumed to treat hepatitis (2). Previous studies have verified the protective effect of a water extract of *L. microphyllum* leaves against carbon tetrachloride-induced hepatotoxicity in rats. Building on these findings, the present investigation used a different hepatotoxic agent, a high dose of paracetamol, to evaluate the efficacy of the plant’s ethyl acetate extract.

Paracetamol, or acetaminophen, is among the most widely used over-the-counter medications globally for managing pain and fever. Its therapeutic benefits are achieved within a defined dose range; however, excessive or prolonged intake can induce significant hepatotoxicity (3). At standard doses, paracetamol is metabolized primarily through hepatic glucuronidation and sulfation. A minor fraction undergoes cytochrome P450-mediated oxidation, yielding the reactive intermediate N-acetyl-p-benzoquinone imine (NAPQI). Under normal conditions, NAPQI is rapidly detoxified by conjugation with hepatic glutathione (GSH) and excreted. In overdose, the primary metabolic pathways become saturated, leading to NAPQI accumulation. This accumulation depletes glutathione reserves and causes oxidative stress, ultimately resulting in hepatocellular damage (3).

In experimental models, administration of paracetamol at 3 g/kg body weight for 7 days induces liver injury, marked by elevated serum levels of AST and ALT (4). The current study used ethyl acetate for extraction because this solvent yielded an extract with notable antioxidant activity (IC_50_ = 17.39 mg/L). The observed hepatoprotective effect is attributed, in part, to the presence of quercetin, a flavonoid identified in the extract (5, 6).

## 2. Objectives

This study aimed to evaluate the in vivo hepatoprotective efficacy and optimal dose of EALM in Wistar rats with paracetamol-induced hepatic injury.

## 3. Methods

### 3.1. Plant Materials and Extraction

Leaves of *L. microphyllum* were collected and identified, and a voucher specimen (750IPH101) was deposited at the Herbarium Bogoriense, Research Centre for Biology, Indonesian Institute of Sciences. The plant material was taxonomically authenticated as *Lygodium microphyllum* (Cav.) R.Br. (family Lygodiaceae) by a certified botanist, Dr. Atik Retnowati. The collected material was cleaned, air-dried, and then oven-dried at 50°C for 48 hours.

**Figure 1.**
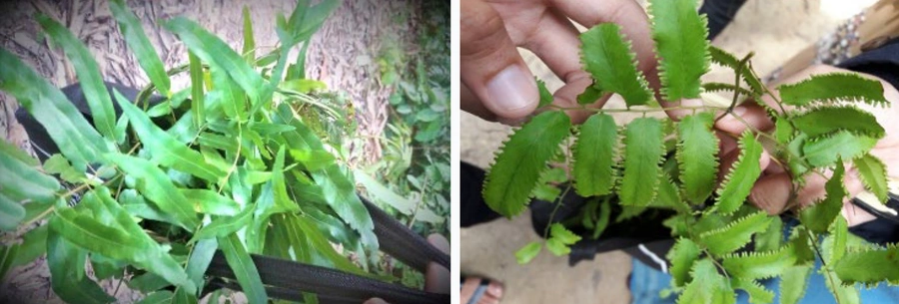
Images of *L. microphyllum* leaves (fertile and infertile)

A sequential maceration procedure was used. The dried powder was first extracted with n-hexane for 72 hours at room temperature, filtered, and concentrated using a rotary evaporator at 50°C. The residual plant material was dried to remove residual hexane and subsequently re-macerated in ethyl acetate under identical conditions. The ethyl acetate filtrate was evaporated to yield the final EALM, which was stored for further analysis (Figure 1).

### 3.2. Experimental Animals

Twenty-four male Wistar rats (200 - 250 g) were used in this pilot exploratory in vivo study (7). The design aimed to identify preliminary biological trends and estimate effect magnitudes rather than test definitive hypotheses. Animals were housed under standard laboratory conditions at 25°C with a 12-hour light/dark cycle and provided ad libitum access to food and water (8, 9). All procedures complied with the 3Rs principles (Replacement, Reduction, and Refinement), and the data exclusion criteria were established a priori in accordance with the ARRIVE 2.0 guidelines (10). The study was approved by the Health Research Ethics Committee of the Faculty of Medicine, Universitas Mulawarman (Approval No. 78/KEPK-FK/IX/2024). Allocation concealment was implemented by assigning the animals to cages labeled with random numerical codes by an independent laboratory technician who was not involved in data analysis (9).

### 3.3. Assessment of Hepatoprotective Activity

Hepatoprotective evaluation was conducted using a previously established method with modifications. Twenty-four male rats were randomly allocated into six groups: a normal control group (N, receiving 0.5% NaCMC), a paracetamol-induced negative control group (PCT, 3 g/kg BW), and three treatment groups administered EALM at doses of 200, 400, and 600 mg/kg BW (EALM 1 - 3), respectively. The extract was administered orally once daily for 14 consecutive days. Hepatotoxicity was induced on day 8 by oral administration of paracetamol to all groups except the normal control group. On day 15, the animals were euthanized, and blood and liver tissue were collected for analysis. To minimize observer bias, serum biochemical analysis (AST and ALT) and histopathological scoring were performed in a blinded manner, with the observer or analyst unaware of treatment group identity until all data were collected (9).

### 3.4. Measurement of AST and ALT Levels

Aspartate aminotransferase and ALT activities were determined as previously described. Blood was collected longitudinally from each animal on days 0, 8, and 15. Minimally invasive tail vein sampling (0.5 - 1 mL) was performed on days 0 and 8, whereas terminal blood collection was conducted via the vena cava on day 15. Plasma was separated by centrifugation at 3000 rpm for 10 minutes and analyzed for AST and ALT activity using a Mindray BS-300 Chemistry Analyzer according to the manufacturer's protocol. Results are expressed in U/L.

### 3.5. Histopathological Examination

Liver tissues were fixed in 10% buffered formalin, dehydrated through a graded ethanol series, cleared in xylene, and embedded in paraffin. Sections of 5 μm thickness were prepared, stained with hematoxylin and eosin (H&E), and examined under a light microscope to evaluate histopathological changes. Liver tissue damage was evaluated using a standard 0 - 4 scoring scale adapted from Gibson-Corley et al. (2013): score 0, no lesion/normal; score 1, minimal damage (< 25% of the affected area); score 2, mild damage (25% - 50% of the affected area); score 3, moderate damage (50% - 75% of the affected area); and score 4, severe/extensive damage (> 75% of the affected area) (11). Five fields per slide were selected using systematic random sampling in the centrilobular area (Zone 3), because paracetamol specifically induces necrosis in this region due to high concentrations of cytochrome P450 enzymes (12).

## 4. Results

### 4.1. Extraction Yield

The extract was obtained by sequential maceration with ethyl acetate after initial defatting with n-hexane. The final yield was 4% (w/w), corresponding to 4 g of dried extract obtained from 100 g of dried leaf material. This yield represents a concentrated fraction of medium-polarity phytoconstituents, including flavonoids and phenolic compounds, which are commonly associated with antioxidant and hepatoprotective activities.

### 4.2. Clinical Observations: Body Weight Monitoring

Liver dysfunction disrupts protein synthesis and metabolic homeostasis, often manifesting as systemic catabolism and weight loss (13, 14). As a general indicator of overall health, body weight was recorded daily in all animals throughout the 14-day experimental period. A clear divergence in weight trajectory was observed after paracetamol induction on day 8. Rats in the negative control group (paracetamol only) exhibited a significant and progressive decline in body mass beginning 48 hours after induction. This finding is a classical clinical sign of hepatic failure and metabolic dysregulation.

**Figure 2.**
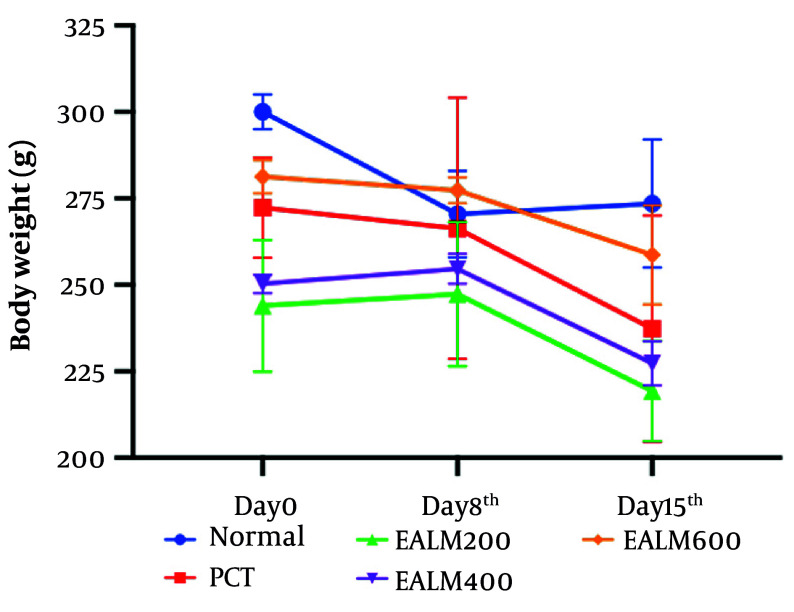
Effects of EALM on the body weight of hepatotoxic rats given paracetamol. Body weight was examined using two-way analysis of variance. Each point on the graph represents the mean ± SD of four rats in each group.

In contrast, animals in the normal control group maintained steady weight gain. Groups pretreated with EALM at doses of 200, 400, and 600 mg/kg BW showed no significant alteration in their growth curves before induction, confirming the lack of intrinsic toxicity of the extract. After the paracetamol challenge, these treated groups showed a markedly attenuated weight-loss profile compared with the negative control group. The group receiving 200 mg/kg BW showed the most favorable recovery trend, with weight stabilizing and beginning to increase by the study endpoint. These clinical observations, summarized in Figure 2, provide supportive evidence that the hepatoprotective effect of EALM translates into a measurable systemic benefit by preserving metabolic function (14).

### 4.3. Biochemical Markers: Serum Transaminase Levels

Hepatocyte membrane integrity was quantitatively assessed by measuring the leakage of the cytosolic enzymes AST and ALT into the bloodstream. Blood samples were collected at three critical intervals: day 0 (baseline), day 8 (after 1 week of prophylactic extract administration but before paracetamol induction), and day 15 (1 week after induction). Analysis of samples from day 8 revealed no statistically significant elevation in serum AST or ALT in any EALM-treated group compared with baseline. This finding indicates that the extract, at the administered doses, does not induce subclinical hepatotoxicity or stress.

**Figure 3.**
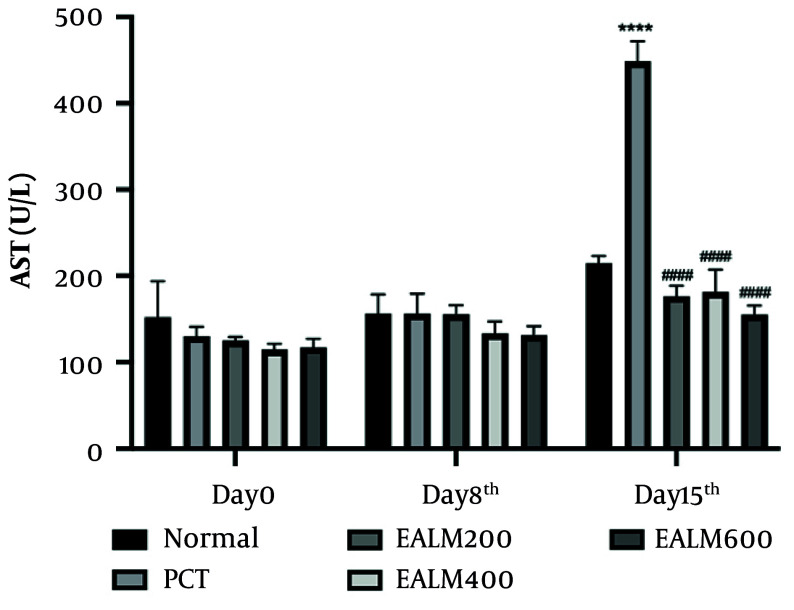
Effects of EALM on AST levels in paracetamol-induced hepatotoxic rats. AST levels were examined using two-way analysis of variance. Each bar represents the mean ± SD of four rats in each group. #### P < 0.0001 compared with the paracetamol group (PCT); **** P < 0.0001 compared with the normal group.

**Figure 4.**
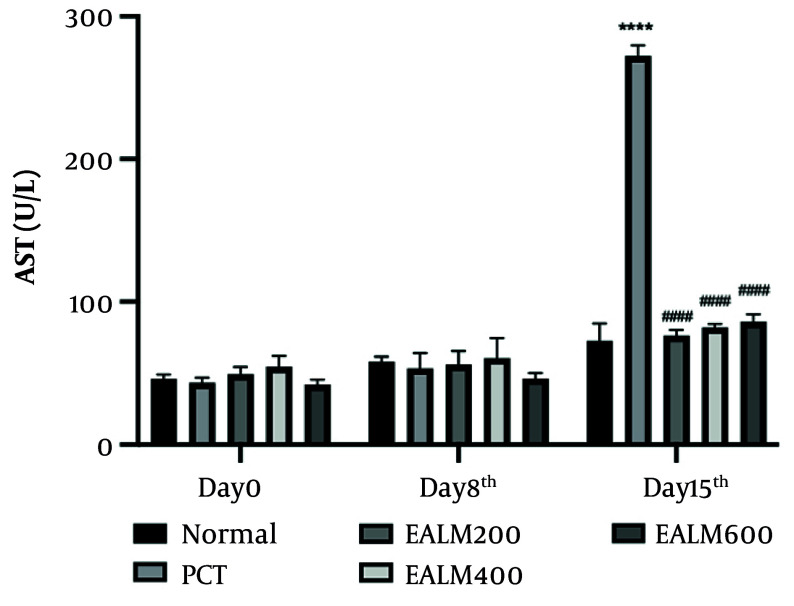
Effects of EALM on ALT levels in paracetamol-induced hepatotoxic rats. ALT levels were examined using two-way analysis of variance. Each bar represents the mean ± SD of four rats in each group. #### P < 0.0001 compared with the paracetamol group (PCT); **** P < 0.0001 compared with the normal group.

The results from day 15 were definitive. Statistical analysis using analysis of variance followed by Tukey's honestly significant difference test showed a highly significant surge in both enzyme markers in the paracetamol-only group (P < 0.001), confirming severe hepatotoxicity (15). All groups pretreated with EALM exhibited a statistically significant reduction in this enzyme elevation. A clear dose-response relationship was evident. The cohort receiving 200 mg/kg BW EALM presented the most favorable biochemical profile; their postinduction ALT and AST values showed the smallest absolute increase and were closest to established normal reference ranges for healthy rats (approximately 41 U/L for ALT and 152 U/L for AST) (15) (Figures 3 and 4).

### 4.4. Histopathological Findings

Histological evaluation of liver tissue provides essential morphological confirmation of the biochemical findings. Processed liver sections were stained with H&E and examined microscopically at 400 × magnification. A semiquantitative scoring system was applied to assess key lesions, including hydropic degeneration and hepatocellular necrosis, across five representative fields per sample (11).

**Figure 5.**
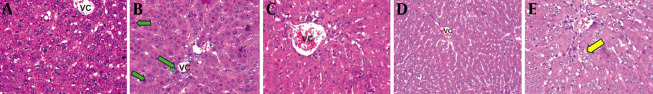
Effect of EALM on liver tissue histopathology in rats given paracetamol to induce hepatotoxicity. (A) Normal group; (B) paracetamol group; (C) EALM 200 mg/kg BW; (D) EALM 400 mg/kg BW; and (E) EALM 600 mg/kg BW at 400 × magnification. Green arrows indicate pyknosis, and yellow arrows indicate degeneration.

**Figure 6.**
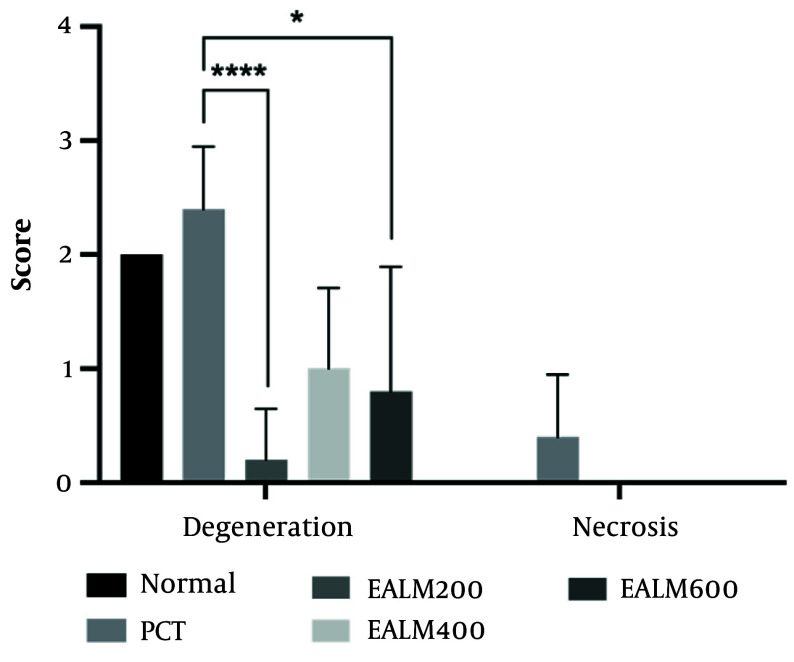
Histopathology liver score for the effects of EALM on degeneration and necrosis in hepatotoxic rats caused by paracetamol. Histopathology scores were analyzed using one-way analysis of variance. Each bar represents the mean ± SD of three rats in each group. **** P < 0.0001; *** P < 0.001; and * P < 0.05.

The histopathological results provided conclusive morphological support. Liver sections from the negative control group displayed extensive damage, including widespread marked hydropic degeneration, nuclear pyknosis, and multiple necrotic foci. All groups pretreated with EALM demonstrated substantial histological preservation. The most pronounced protection was observed in the 200 mg/kg BW group, in which liver architecture was virtually normal, with only minimal focal hydropic changes and a complete absence of necrosis. The 400 and 600 mg/kg BW groups also showed significant protection, with mild hydropic degeneration but no necrosis (Figures 5 and 6).

## 5. Discussion

The primary objective of this study was to evaluate the hepatoprotective potential of EALM against paracetamol-induced acute liver injury in Wistar rats. The clinical, biochemical, and histopathological findings collectively demonstrated a significant protective effect, suggesting that the extract has potential as a botanical intervention against drug-induced hepatotoxicity (13).

The specificity of ALT as a marker primarily of hepatic parenchymal injury strengthens the interpretation of a liver-specific protective effect, because AST is also present in cardiac and muscle tissue (16). The concurrent attenuation of both enzymes, particularly ALT, provides robust biochemical evidence of hepatoprotection. The mechanism underlying this protection is closely linked to the phytochemical profile of the extract. Quercetin, a major flavonoid identified in the extract, is a well-documented bioactive compound with pleiotropic effects. Its hepatoprotective actions are mediated through a synergistic network of antioxidant pathways: 1) direct free-radical scavenging; 2) upregulation of endogenous antioxidant defenses through the Nrf2 pathway, leading to increased glutathione synthesis; 3) attenuation of pro-inflammatory NF-κB signaling; and 4) induction of cytoprotective enzymes, such as heme oxygenase-1 (HO-1) (17-20). By enhancing cellular redox capacity and suppressing inflammatory mediators, these actions directly counteract the primary pathogenetic processes of paracetamol toxicity, including glutathione depletion, oxidative stress, mitochondrial dysfunction, and necrotic cell death (21-23).

Normal hepatic architecture comprises orderly cords of hepatocytes radiating from a central vein, with cells exhibiting distinct vesicular nuclei and eosinophilic cytoplasm (24). The pathological cascade induced by paracetamol involves NAPQI formation, glutathione depletion, protein adduct formation, and ultimately centrilobular necrosis, characterized by cell swelling, nuclear condensation (pyknosis), and cell death (25).

Notably, the normal control group exhibited sporadic, mild hydropic changes. This is a recognized incidental finding in laboratory rodents and is not considered indicative of pathological injury, highlighting the importance of using the intoxicated negative control as the true benchmark for damage assessment (26-28). The observed hydropic degeneration is mechanistically linked to oxidative inhibition of the Na^+^/K^+^-ATPase pump, leading to intracellular ion imbalance and water accumulation (29).

### 5.1. Conclusions

The convergent evidence from this study demonstrates that EALM confers significant, dose-dependent hepatoprotection against paracetamol-induced liver injury in Wistar rats. Although all tested doses (200, 400, and 600 mg/kg BW) provided substantial protection, the 200 mg/kg BW dose consistently emerged as the most efficacious. This dose yielded optimal outcomes across all parameters, including the best-preserved clinical status (body weight), biochemical function (serum transaminases closest to normal), and cellular morphology (near-normal histology). The efficacy of this lower dose suggests an optimal therapeutic window for the active constituents. Therefore, EALM, particularly at a dose of 200 mg/kg BW, represents a promising candidate for further investigation as a natural prophylactic or adjunctive agent against chemical hepatotoxicity.

## Data Availability

The dataset presented in the study is available on request from the corresponding author during submission or after publication.
